# Editorial: Emerging swine infectious diseases

**DOI:** 10.3389/fvets.2023.1349844

**Published:** 2023-12-19

**Authors:** Hongchao Gou, Xia Zhou, Shao-Lun Zhai

**Affiliations:** ^1^Institute of Animal Health, Guangdong Academy of Agricultural Sciences, Guangzhou, China; ^2^Guangdong Provincial Key Laboratory of Livestock Disease Prevention, Guangzhou, China; ^3^Scientific Observation and Experiment Station of Veterinary Drugs and Diagnostic Techniques of Guangdong Province, Guangzhou, China

**Keywords:** swine emerging infectious diseases, swine emerging viruses, PCV3, PCV4, swine coronavirus

## 1 Introduction

As major threats to the global swine industry, swine infectious diseases caused significant economic losses and potential public health issues. During the past three decades, many swine infectious diseases emerged in the field, such as porcine reproductive and respiratory syndrome virus (PRRSV) and its novel isolates with distinct pathogenicity, high pathogenic variants of porcine epidemic diarrhea virus (PEDV) and pseudorabies virus (PRV) and influenza viruses, which lead to tremendous economic losses worldwide. A few novel pathogens identified recently, such as Senecavirus A, atypical porcine pestivirus (APPV), porcine circovirus 3 (PCV3), porcine circovirus 4 (PCV4), porcine deltacoronavirus (PDCoV), swine acute diarrhea syndrome coronavirus (SADS-CoV), influenza D virus (IDV), constitute a new challenge (1–5). This Research Topic is focused on filling in some gaps of emerging swine infectious diseases from diverse aspects, such as the pathogenesis mechanisms, structure and function of viral proteins, protective immunity, viral evolution, and new approaches for prevention and treatment.

## 2 Organization of the Research Topic

In this Research Topic, we received 20 manuscripts, nine (seven original research, one perspective, one brief research report) were accepted for publication. Among them, nine papers all involved virus research. Stelder et al. designed an experiment in Romania to quantify which species of mosquitoes are attracted to Romanian backyard pigs, which species take blood meals from these, and whether these observed feeding behaviors vary throughout the vector season. PRRSV has caused huge economic losses for the global pig industry, but its origins and evolution remain a mystery. According to the genome sequences of seven arteriviruses isolated from rodents in 2018, Zhao et al. offered new analysis showing that they may be ancestors of PRRSV. Sanchez et al. used a spatial and spatiotemporal kernel density approach to estimate PRRSV relative risk and utilized a Bayesian spatiotemporal hierarchical model to assess the effects of environmental variables, between-farm movement data and on-farm biosecurity features on PRRSV outbreaks in the United States.

In order to effectively monitor swine coronaviruses, a quadruplex reverse transcription-polymerase chain reaction (RT-PCR) method for the simultaneous detection of PEDV, PDCoV, TGEV and SADS-CoV was developed by Niu et al., and TaqMan probe-based multiplex real-time quantitative reverse transcription-polymerase chain reaction (qRT-PCR) was developed by Li et al.. By systematical analysis of all available full-length genomes of TGEVs (n = 43) and porcine respiratory coronaviruses PRCVs (*n* = 7), Wang et al. showed that TGEVs fell into two independent evolutionary phylogenetic clades, GI and GII. Viruses circulating in China (until 2021) clustered with the traditional or attenuated vaccine strains within the same evolutionary clades (GI). To study the cross-reaction and cross-neutralizing activities of antibodies against different genotypes (G) of E2 glycoproteins, ectodomains of G1.1, G2.1, G2.1d, and G3.4 CSFV E2 glycoproteins from a mammalian cell expression system were generated by Chen et al.. To evaluate the virulence reversion potential risk, rPRRSV-E2 had been continuously passaged *in vivo*, the stability of E2 expression and virulence of the passage viruses were analyzed by Jiang et al.. Sirisereewan et al. investigated the genetic diversity of PCV2 strains circulating in Thailand between 2019 and 2020 using 742 swine clinical samples from 145 farms.

## 3 Conclusion

Since the July of 2022, The Research Topic began to receive the manuscript submission, and invited more than 24 research teams from the world to submit the manuscript, we finally received 20 manuscripts. Although the Topic provides overviews of some swine emerging pathogen and novel strategies for the detection and control, the manuscripts associated with swine emerging pathogen (especially PCV3, PCV4, IDV) were lacking. In the following work, we hope that more scientists pay more attention to emerging swine infectious diseases.

## Author contributions

HG: Writing—original draft. XZ: Writing—original draft. S-LZ: Writing—original draft, Writing—review & editing.
